# Supplementary material for: Mapping the evolution of fertility support policies in China: A content and instrumental analysis
Source: PLoS One. 2025 Oct 9;20(10):e0332137. doi: 10.1371/journal.pone.0332137 (PMC12510515; doi:10.1371/journal.pone.0332137)
Supplement: S1 Appendix — (ZIP) [file pone.0332137.s001.zip › S1 Appendix. 226 original policy documents/20-国家卫生计生委办公厅关于印发2015年计划生育宣传工作提纲的通知(FBM-CLI-4-246615).doc]

国家卫生计生委办公厅关于印发2015年计划生育宣传工作提纲的通知

**发布部门：** [国家卫生和计划生育委员会(已撤销)](https://www.pkulaw.com/law/chl?Aggs.IssueDepartment=60319) [机构沿革](javascript:void(0))

**发文字号：**国卫办指导函〔2015〕243号

**发布日期：**2015.03.27

**实施日期：**2015.03.27

**时效性：** [现行有效](https://www.pkulaw.com/law/chl?Aggs.TimelinessDic=01)

**效力级别：** [部门工作文件](https://www.pkulaw.com/law/chl?Aggs.EffectivenessDic=XE0303)

**法规类别：** [计划生育管理](https://www.pkulaw.com/law/chl?Aggs.Category=09705)

国家卫生计生委办公厅关于印发2015年计划生育宣传工作提纲的通知
（国卫办指导函〔2015〕243号）

各省、自治区、直辖市卫生计生委，新疆生产建设兵团人口计生委：
　　为进一步做好计划生育宣传工作，我委研究制定了2015年计划生育宣传工作提纲。现印发给你们，请结合地方实际，遵照执行。

国家卫生计生委办公厅
2015年3月27日

　　2015年计划生育宣传工作提纲

　　2015年计划生育宣传工作的总体要求是：深入贯彻党的十八大和十八届三中、四中全会精神以及《中共中央　国务院关于调整完善生育政策的意见》（中发〔2013〕15号）精神，紧紧围绕卫生计生事业发展大局和计划生育工作重点任务，坚持“围绕中心、服务大局、突出重点、正面引导”的原则，充分发挥宣传教育先行先导作用，为落实计划生育基本国策、逐步调整完善生育政策、促进人口长期均衡发展提供思想保证、精神动力、舆论支持和文化条件。
　　**一、**深入宣传计划生育基本国策
　　深入宣传我国人口国情、计划生育基本国策，宣传计划生育工作取得的巨大成就，宣传计划生育男女平等、优生优育的文明生育观念，深化婚育新风进万家活动，增强全社会的国情、国策意识。

　　**二、**加强单独两孩政策宣传
　　大力宣传调整完善生育政策的重要意义，宣传单独两孩政策实施以来取得的阶段性成效，宣传计划生育服务管理改革成果。推出一批典型经验和特色报道，做好信息公开和政策解读，积极回应社会关切。

　　**三、**大力宣传先进典型
　　组织开展最美基层计划生育工作者评选宣传活动。通过先进模范人物评选表彰、先进事迹巡回报告、开发制作影视作品等多种形式，树立一批计划生育先进典型，弘扬主旋律，传递正能量。

　　**四、**精心组织理论宣传
　　借助人口学会等平台和专家力量，组织开展人口与计划生育工作理论研究，加大理论宣传力度，刊发有深度的理论文章，推出有分量的研究成果。编辑出版计划生育理论宣传读物，提高全社会对计划生育工作重要性的认识。

　　**五、**积极推进宣传阵地建设
　　搭建主流媒体计划生育宣传平台，增强计划生育宣传的公信力和影响力。充分发挥各级卫生计生行政部门官方网站、微博、微信的作用，及时发布权威信息。推进“人口文化大院”建设。结合实际开发宣传品、开展宣传活动。

　　**六、**着力优化户外宣传环境
　　在城乡主要街道、文化广场、村（居）小区等场所，设置内容温馨、形式美观的宣传橱窗、宣传栏、电子荧屏、电子灯箱等，清理过时陈旧、破损残缺的宣传标语，刷写适应形势、语言温馨、通俗易懂的宣传标语。

　　**七、**加大舆情监测力度
　　树立“守土有责”意识，重点加强对网上言论的正面引导，提高舆论引导的有效性、及时性，妥善应对负面炒作。对恶意攻击计划生育基本国策的错误言论，要会同有关部门及时处置，努力营造良好的舆论氛围。

　　**八、**切实加强组织领导
　　坚持把计划生育宣传工作摆上重要位置，纳入目标管理责任制考核，建立“一把手”负总责，分管领导具体抓的领导责任制。各级卫生计生宣传部门组织实施，基层指导及相关部门密切配合。
　　附件：

2015年计划生育宣传标语

　　1.坚持计划生育基本国策，促进人口长期均衡发展。
　　2.完善生育政策，利国利民利家。
　　3.计生国策心系群众，奖励扶助情暖人心。
　　4.知情选择，科学孕育。
　　5.幸福生活从优生优育开始。
　　6.婚育新风进万家，优生快富奔小康。
　　7.优生优育优教，利国利民利家。
　　8.按政策生育，负责任养育。
　　9.弘扬家庭美德，塑造良好家风。

© 北大法宝：（[www.pkulaw.com](https://www.pkulaw.com/)) 专业提供法律信息、法学知识和法律软件领域各类解决方案。北大法宝为您提供丰富的参考资料，正式引用法规条文时请与标准文本核对。
欢迎查看所有[产品和服务](https://www.pkulaw.com/helps/2.html)。法宝快讯：[如何快速找到您需要的检索结果？](https://www.pkulaw.com/helps/70.html)[法宝](https://www.pkulaw.com/helps/69.html)V6有何新特色？

扫描二维码阅读原文

**原文链接：**<https://www.pkulaw.com/chl/cbea3bc41270ed5ebdfb.html>
